# Determinants and Patterns of Reproductive Success in the Greater Horseshoe Bat during a Population Recovery

**DOI:** 10.1371/journal.pone.0087199

**Published:** 2014-02-13

**Authors:** Helen L. Ward, Roger D. Ransome, Gareth Jones, Stephen J. Rossiter

**Affiliations:** 1 School of Biological and Chemical Sciences, Queen Mary University of London, London, United Kingdom; 2 School of Biological Sciences, University of Bristol, Bristol, United Kingdom; Università degli Studi di Napoli Federico II, Italy

## Abstract

An individual's reproductive success will depend on traits that increase access to mates, as well as the number of mates available. In most well-studied mammals, males are the larger sex, and body size often increases success in intra-sexual contests and thus paternity. In comparison, the determinants of male success in species with reversed sexual size dimorphism (RSD) are less well understood. Greater horseshoe bats (*Rhinolophus ferrumequinum*) exhibit RSD and females appear to exert mate choice when they visit and copulate with males in their underground territories. Here we assessed putative determinants of reproductive success in a colony of greater horseshoe bats during a 19-year period of rapid population growth. We genotyped 1080 bats with up to 40 microsatellite loci and assigned maternity to 99.5% of pups, and paternity to 76.8% of pups. We found that in spite of RSD, paternity success correlated positively with male size, and, consistent with our previous findings, also with age. Female reproductive success, which has not previously been studied in this population, was also age-related and correlated positively with individual heterozygosity, but not with body size. Remarkable male reproductive skew was detected that initially increased steadily with population size, possibly coinciding with the saturation of suitable territories, but then levelled off suggesting an upper limit to a male's number of partners. Our results illustrate that RSD can occur alongside intense male sexual competition, that male breeding success is density-dependent, and that male and female greater horseshoe bats are subject to different selective pressures.

## Introduction

Most studies of sexual selection in mammals have focused on species that show male-biased size dimorphism. Where males compete with each other for access to females, large body size often confers an advantage and thus correlates positively with individual paternity success [1]. High variance in breeding success among individuals leads to reproductive skew at the population level, which has been shown in a range of mammalian groups including ungulates [2], primates [3], pinnipeds [4], carnivores [5] and bats [6]. In addition to size, other sources of variation (some correlated with size) may also influence a male's access to females, such as whether he holds a high quality territory [7], his age [8] and his level of heterozygosity [9].

Much less well studied are the determinants of reproductive skew in species where females are the larger sex. In vertebrates, such so-called reversed sexual size dimorphism (RSD) occurs in diverse taxa, including birds, fishes, anurans [10], [11], [12] and, in mammals, in groups such as rodents, primates, ungulates and bats [13]. Despite this, few genetic analyses of breeding success in mammals with RSD have been conducted, and the results have been mixed. For example, while taxa such as the yellow-pine chipmunk (*Tamias amoenus*) [14] exhibit low variance in paternity success, others, such as the spotted hyena (*Crocuta crocuta*), show stronger polygyny [15]. Hence the determinants of male success in taxa with RSD are not always easily predictable. One theory is that RSD might evolve where smaller males have a competitive advantage either due to enhanced agility in sexual contests or because of female preferences for some aspect of mobility (e.g. [16]), a trend that has support from both invertebrates and vertebrates [17], [18], [19]. On the other hand, large male garter snakes *Thamnophis sirtalis* gain more mating success than their smaller rivals, which are physically displaced from females [20], while in female meerkats (*Suricata suricatta*), large body size enhances reproductive success [21]. Undoubtedly, clear associations between body size and reproductive success might be obscured, reduced or even absent in cases where body size is under strong natural selection; for example, large body mass might increase survival [22], [23], or for females may reduce the proportional load of carrying young [24].

Regardless of phenotypic attributes, individual reproductive success critically depends on the availability of partners. Simulations of male-male competition and female choice predict that high female densities will further enhance the success of polygynous males and so increase overall paternity skew, whereas at lower densities or smaller populations, sexual selection and sexual conflict will be less intense [25]. Empirical findings, however, are more equivocal; several studies suggest skew might actually fall at high densities under some conditions, for example, due to the difficulties of defending resources [25], [26]. These and similar mixed results highlight a need for more investigations of the relationship between sexual selection and population density in natural populations.

Deep insights into vertebrate breeding systems and sexual selection have often come from long-term studies of natural populations for which longitudinal pedigree data are available and thus reproductive success has been measured over individual lifetimes. Examples of such studies are relatively rare and mostly restricted to birds and mammals, especially passerines [27], [28] [29], ungulates [30] [31], [32] and primates [33], [34]. The population of greater horseshoe bats *Rhinolophus ferrumequinum* that roosts in and around Woodchester Mansion in southwest England has been studied intensively since the late 1950s [35], [36], [37], [38] with genetic material collected since 1993. This species shows clear RSD; males are approximately 2% smaller (based on forearm length) and 2–15% lighter than females [39]. Previous studies of paternity success across 10 years (1993–2002) documented significant long term reproductive skew within the population and demonstrated that annual male paternity success is age-related [38], [40]. However, marked variance in reproductive success among males of the same age pointed to additional unidentified determinants of fitness. Anecdotal evidence from one successful male suggested small size may be an advantage [40], yet limited sample sizes precluded a formal test of this association. If small body size does indeed confer a fitness advantage in this species, then this raises questions about how males might defend their mating territories from other males.

During the past nine years since our previous parentage analysis was conducted, the Woodchester population has undergone a period of rapid growth, with 96% more offspring (n = 92) born in 2011 than in 2002, and 283% more than in 1993. This almost monotonic year-on-year increase in numbers began in 1988 following a population crash caused by exceptionally poor weather conditions in the previous four winters that continued into spring and mirrors the demographic trend seen more widely in this species across the UK [36]. Samples and data collected during this timeframe provide an opportunity to elucidate the factors influencing reproductive output in a species with RSD, as well as characterise the patterns of annual and long-term skew during a 19-year period of rapid population growth, when competition for territories and thus access to mates among males is expected to have intensified. Here we address these questions by combining microsatellite-based parentage inference with linear modelling of potential phenotypic and genetic fitness determinants. Specifically, we first explore whether the association between paternity success and body size is negative, as seen in some volant taxa with RSD, or positive, as might be more expected from the system of resource-defence polygyny observed in greater horseshoe bats. We hypothesized that reproductive success of resource-holding males, and thus overall paternity skew, would increase with population size, reflecting the higher availability of females. On the other hand we expected that most females would breed each year and thus breeding skew among females would remain consistently low across the study period with little or no effect of phenotypic factors.

## Materials and Methods

### Ethics statement

Greater horseshoe bats are a protected species in the U.K. and bats were caught and sampled under licences from English Nature and the Home Office (PPL 30/2513). Permission for access to catch bats at the maternity roost was granted by the Woodchester Mansion Trust, and for access to catch bats in caves and mines on land that is privately owned, from the land owners.

### Study site and background

This study focused on a maternity colony that assembles each summer in the attic of Woodchester Mansion, Gloucestershire, U.K. (51°43′N, 2°18′W). Greater horseshoe bats have been caught and ringed at Woodchester Mansion for 54 years, and tissue samples have been collected annually from all offspring born into the colony since 1993 as well as their mothers. Primary catches, during which new-born pups are ringed and sampled, take place in early to mid-July, at which time most pups are less than 20 days old and still attached to their mothers. July catches also afford an opportunity to sample any adult females in the breeding colony that have not been previously sampled or ringed.

In addition to catches at Woodchester Mansion, surveys of all known caves and mines – used as mating sites and hibernacula – within a 25 km radius of the maternity roost are performed during autumn, winter and spring as part of an on-going study of this population [35], [36], [41]. During these surveys, greater horseshoe bats present are recorded and, where necessary, ringed and tissue sampled. Immigrant adults, especially males, in the population are usually found during these surveys. Based on current recapture rates, we estimate that over 90% of bats in this population have been ringed, including all breeding females [38].

A single tissue sample is taken from the uropatagium (tail-membrane) of each bat using a sterile 3 mm skin biopsy punch (Miltex). These are placed into individually labelled tubes containing absolute (>99%) ethanol, and stored in a −20°C freezer. Measurements taken from each individual include sex, length of forearm (radius), length of the 5^th^ digit and, if relevant, the ring number of the female to which the juvenile was found attached. Date of birth is also recorded for new-borns, or estimated from forearm length for older juveniles (see Rossiter *et al.* [42] for further details of these measurements). Bats caught that have been previously sampled are not resampled; repeat physical measurements, however, are taken. By 2012 1,080 individual bats had been sampled.

### DNA isolation and microsatellite genotyping

Genomic DNA was extracted from tissue samples using DNeasy kits (QIAGEN) as per the manufacturer's instructions. Prior to this work, primers for amplifying 21 polymorphic microsatellite loci, developed specifically for *Rhinolophus ferrumequinum* [43], [44], had been used to genotype 454 individual bats sampled between 1993 and 2002 from the Woodchester population (Rossiter *et al.* [38]). Here we increased this suite of microsatellite loci by optimising primers developed for four congeneric species [45], [46], [47]. At the same time we discarded 7 of the original microsatellite loci on the basis of poor amplification results in comparison to new loci. This gave us 33 loci in total.

All 33 loci were amplified in 741 individuals that have been sampled from the Woodchester Mansion population since 2003 and kept in storage. Following completion of this task, we also genotyped 352 of the 454 individuals sampled before 2003 for which we still had ample DNA at the 19 new available loci. Hence, upon completion of genotyping, 102 individual samples were genotyped at up to 21 loci (by Rossiter *et al.* [38]), 352 individual samples at up to 40 loci (the 33 loci used in this study plus the seven loci discarded but for which data are available from earlier genotyping by Rossiter *et al.* [38], and 741 samples at up to 33 loci. In reality, many of the pre-2003 individuals are genotyped at more loci than stated above because they were resampled post-2003 and the second sample was genotyped for newly developed loci. All 40 microsatellites and their characteristics when amplified in the Woodchester Mansion population of greater horseshoe bats, along with accession numbers (GenBank), are presented in Table S1.

All primers were 5′ fluoro-labelled and 10 µL PCR reactions contained 5 µL QIAGEN multiplex PCR Master Mix (HotStarTaq DNA Polymerase, dNTP mix, Multiplex PCR buffer containing 3 mM MgCl_2_), variable volumes of primer mix depending on how many primers were in the multiplex (each primer was at a final concentration of 0.2 µM) and 1.5 µL of 20–30 ng/µL DNA. The remaining volume was made up with MilliQ water. PCRs were performed on a DNA Engine Tetrad®Thermal Cycler (MJ Research) and PCR profiles included a denaturation step of 95°C for 300 s, then 28 cycles of 95°C for 30 s, specific annealing temperature for 90 s, 72°C for 90 s, before a final extension of 60°C for 30 min. PCR products were diluted by a factor of 150 with MilliQ water, and 2 µL of this mixture was added to a size standard (0.08 µL GeneScan™ 400HD ROX™ Size Standard with 7.92 µL Applied Biosystems Hi-Di Formamide) then heated for 300 s at 95°C before fragment lengths could be visualised using capillary electrophoresis (Applied Biosystems 3730 DNA analyser). Genotypes were analysed and assigned using GeneMapper version 3.7; all electropherograms were checked by eye.

Allele frequencies, deviations from Hardy-Weinberg equilibrium, and estimates of null allele frequencies were estimated for each locus using genotype data from all individuals in cervus v 3.0 [48].

### Maternity analysis

To perform parentage analyses we used the maximum likelihood approach implemented in cervus [48]. cervus calculates the log-likelihood of each candidate parent being the true parent relative to an arbitrary individual and then calculates the difference between the two most likely parents (Delta, Δ). Critical values of Δ are determined by computer simulation, which incorporates a realistic rate of sampling error and also removes a specified proportion of candidate parents to reflect the real world in which not all animals are sampled. Since greater horseshoe bat mothers appear to only suckle their own offspring [40], in almost all cases, mother-pup attachment observations could be used to assign maternity. However, to confirm these relationships, we used cervus to complete maternity analyses, by cohort, from 1993 to 2011, with a default error rate of 1%. As mothers only give birth to one pup per year, matched individuals could be eliminated from the pool of candidate mothers until all mother–pup pairs, including those that were unattached at the time of catching, were identified. In years except 2010, simulations for deriving confidence estimates were performed assuming a 100% sampling parameter as all candidate mothers were found attached to infants during surveys of the breeding colony. The 2010 simulation was conducted with a sampling parameter of 88% because not all candidate mothers were established in this year as several offspring were found unattached and not in close proximity to adult females. If a female was found attached to a pup in the attic, it was assigned as the mother of that pup if it had no more than two genetic mismatches, and the pair confidence was greater than or equal to 95%. If a female was not found attached to a pup, but was suggested as the mother of that pup, it was assigned if it had the top delta value and no more than two genetic mismatches when at least 10 loci were compared, and the pair confidence was greater than or equal to 95%. The combined probability of exclusion ranged from 0.99920 when the genotypes of a candidate mother and an offspring were compared at the minimum number of loci (10), to 0.99995 at the maximum number of loci (38).

### Paternity analysis

Following successful maternity assignment, unambiguous mother-young pairs were used to perform paternity inference of all pups by cohort. Candidate fathers were assigned in the context of the most likely familial trios, rather than father-pup pairs. A list of candidate fathers was prepared for each cohort, including all males of breeding age (≥2 yrs by the birthing season in question) sampled within the population and believed to be alive during the mating season the preceding autumn. Simulations for deriving confidence estimates were performed assuming a typing error rate of 1% and a sampling rate of 70%; on the basis of recapture rates it is likely a slightly higher proportion of candidate fathers have been sampled, making this is a conservative estimate [40]. A male was assigned paternity to a pup if he had both the highest Delta value at trio level (or pair level if the mother was unknown) and the trio (or pair) confidence was ≥95% when at least 15 loci were compared. A higher minimum number of loci were used in paternity analyses than in maternity analyses as candidate father lists were less refined than candidate mother lists. In addition, the most likely father was only assigned if he had no more than two mismatches with the pup at the pair level, or four mismatches at the trio level, unless the known mother had mismatches with the pup; in which case, the number of mismatches with the known mother, plus four, was allowed. The combined exclusion probability of a candidate father when an offspring's mother was known ranged from 0.97732 for the minimum number of loci (15), to 0.99999 for 38 loci. In seven cases where an offspring's mother remained unknown post-maternity analysis, the combined exclusion probability of a candidate father ranged from 0.99911 (15 loci) to 0.99995 (38 loci).

Following cervus analyses, we used the program colony version 2.0 [49] to resolve outstanding questions of paternity. colony also uses maximum likelihood, however, is also able to use information contained in relationships other than parent-offspring [50]. colony analyses were conducted by cohort and all pups, including those with known parents, were included in analyses together with known maternal and paternal sibships. Candidate parent lists were prepared for cohorts following the method described for cervus. For all analyses, female and male mating systems were classified as polygamous. We used a full-likelihood method, with medium likelihood precision and without a prior.

When assigning parents to pups using colony, only paternity and maternity assignments with a confidence ≥80% were considered following Walling *et al.* [50]. Moreover, an assignment was not accepted if the suggested parent has more than two mismatches with the pup at the pair level, or more than four mismatches at the trio level. Finally, all of the half- and/or full-sibships associated with the focal pup as a result of being assigned to the parent in question had to have a confidence level of greater than or equal to 80%.

### Statistical analyses of reproductive skew

To quantify intrasexual variation in reproductive success within the population, male and female reproductive skew were calculated based on Nonacs's *B* index [51] using the program Skew Calculator [52]. The *B* index measures the observed variance corrected by the expected variance if all individuals were equally successful, with positive values indicating skew, zero indicating a random distribution, and negative values indicating a more even distribution than expected by chance [51]. Significance levels and confidence intervals were estimated by simulation. The *B* index takes into account differences in individual presence, making it suited to studies of wild populations, and allowing levels of skew to be compared across studies [53]. We calculated *B* for each sex for each cohort as well as for all cohorts pooled. When pooled, ‘breeding span’ (the number of years between a breeder's first and last known successful breeding attempts) was used as a measure of presence during the 19 years. Finally we calculated skew among males of the same age. All calculations of skew were based on ‘breeders only’ (individuals that sired at least one pup in the year under consideration). For each *B* index test we used 2000 simulations to assess significance [54].

### Effects of individual age, size and heterozygosity on reproductive success

To assess whether observed inter-individual variation in annual reproductive success among male and female breeders (and thus skew) was explained by differences in age, body size or individual heterozygosity, we developed Generalized Linear Mixed Models (GLMMs) using the packages glmmADMB (Skaug *et al.*, 2012) and lme4 [55] respectively in the software R [56]. Using GLMMs allowed us to have repeated measures from individual animals in the data set by including ‘individual identity’ as a random effect in the models.

Body size was estimated based on forearm length (mm) and individual heterozygosity (Hs_obs) was calculated in the program genhet version 3.1 [57] as (number of heterozygous loci/number of genotyped loci)/mean observed heterozygosity of typed loci. This is a standardized estimate of heterozygosity, sensitive to the fact that sampled individuals might not all be genotyped at the same loci [57]. We built two GLMMs, the first for males and the second for females. In the male model the variable ‘reproductive success’ denoted the number of pups a male sired at a given age and was modelled using a Poisson distribution. Age, forearm length and heterozygosity score were fitted as fixed effects and individual identity and year as random effects, thus controlling for the innate variation among individuals and years, and allowing repeated measures from animals at different ages to be included. Log-likelihood values were used to select the best model. The female model was constructed using the same procedure except that ‘reproductive success’ denoted whether a female had bred or had not bred at a given age, and was thus coded as a binary variable.

Next, to test the effects of size and heterozygosity on an individual's total reproductive success during the 19 year study period, we developed Generalized Linear Models (GLMs) for each sex in the software R, modelling success as a Poisson distribution. Breeding span was included as an independent variable to control for different individuals breeding for periods of different length within the 19 year period. Finally, using the same data set, we developed two more GLMs, one for each sex, to test for differences in the size and heterozygosity of breeding versus non-breeding animals. Breeding status was coded as a binary variable; 1 denoted a breeder and 0 a non-breeder.

## Results

### Parentage analysis

In this study we genotyped 1,080 individual bats at 19–40 microsatellite loci. Two loci, Rferr24 and Rferr25, were discarded post-genotyping as they showed a homozygote excess based on Hardy-Weinberg expectations (χ^2^ = 67.0839, P<0.0001 and χ^2^ = 83.6310, P<0.0001 respectively) over the period 1993–2011. Hence a maximum of 38 loci was used to define the genotype of any one bat during parentage analysis. Having removed Rferr24 and Rferr25 from the data set, the mean number of microsatellite loci genotyped at both alleles in an individual was 32.14 (±SD 4.41), and the mean number of loci genotyped at one or more allele was 32.29 (±SD 4.39).

The number of pups born into the Woodchester Mansion colony each year increased steeply from 24 in 1993 to 92 in 2011 (Table 1). In total, 924 offspring were born into the population during this 19-year period, of which 803 were found attached to an adult female. Mother-pup pairs inferred by attachment were checked for genotypic mismatches and we observed extremely high levels of concurrence between behavioural and genetic maternity assignments, with disagreements in just eight out of 803 (1%) cases. We also assigned mothers to 122 unattached offspring with 95% confidence using cervus, and to two pups with 80% confidence using colony, bringing the total number of maternities assigned to 919 out of 924 (99.5%). Maternity of the five remaining offspring remained unresolved due to low numbers of microsatellite loci successfully genotyped (one case), a single mismatch (two cases), low number of microsatellites successfully genotyped for the candidate mother (one case), or loss of DNA during extraction (one case). All unambiguous mother-young pairs (*n* = 919) were used to infer paternity. We assigned fathers to 703 of the 924 pups at 95% confidence using cervus, and to seven more pups at 80% confidence using colony, giving a total paternity assignment of 710 out of 924 pups (76.8%).

**Table 1:** Paternity assignment and male reproductive skew.

Cohort	*n*	Paternities assigned	No. of sires	Max. no. paternities assigned to one male	Skew (*B* index)
**1993**	24	17 (71%)	10	3	−0.0388
**1994**	25	16 (64%)	10	4	−0.0234
**1995**	28	15 (54%)	10	4	−0.0222
**1996**	32	21 (66%)	13	4	−0.0234
**1997**	29	24 (83%)	12	5	−0.0139
**1998**	33	24 (73%)	11	6	0.0170
**1999**	38	32 (84%)	16	4	−0.0117
**2000**	41	31 (76%)	17	6	−0.0007
**2001**	46	34 (74%)	17	5	−0.0069
**2002**	47	32 (69%)	19	6	−0.0002
**2003**	51	41 (80%)	19	4	−0.0073
**2004**	54	47 (87%)	26	4	−0.0123
**2005**	54	41 (76%)	25	5	−0.0105
**2006**	60	52 (87%)	25	6	−0.0008
**2007**	67	53 (79%)	29	5	−0.0054
**2008**	60	49 (82%)	32	4	−0.0081
**2009**	68	47 (69%)	26	4	−0.0069
**2010**	75	62 (83%)	32	7	−0.0016
**2011**	92	72 (78%)	39	5	−0.0041
**1993–2011**	924	710 (77%)	135	47	0.0047***

Paternity assignment and male reproductive skew based on offspring born into the Woodchester Mansion maternity colony (1993–2011).

*** indicates significance at *P*<0.001, *n* denotes cohort size.

### Male reproductive success

**Figure 1:**
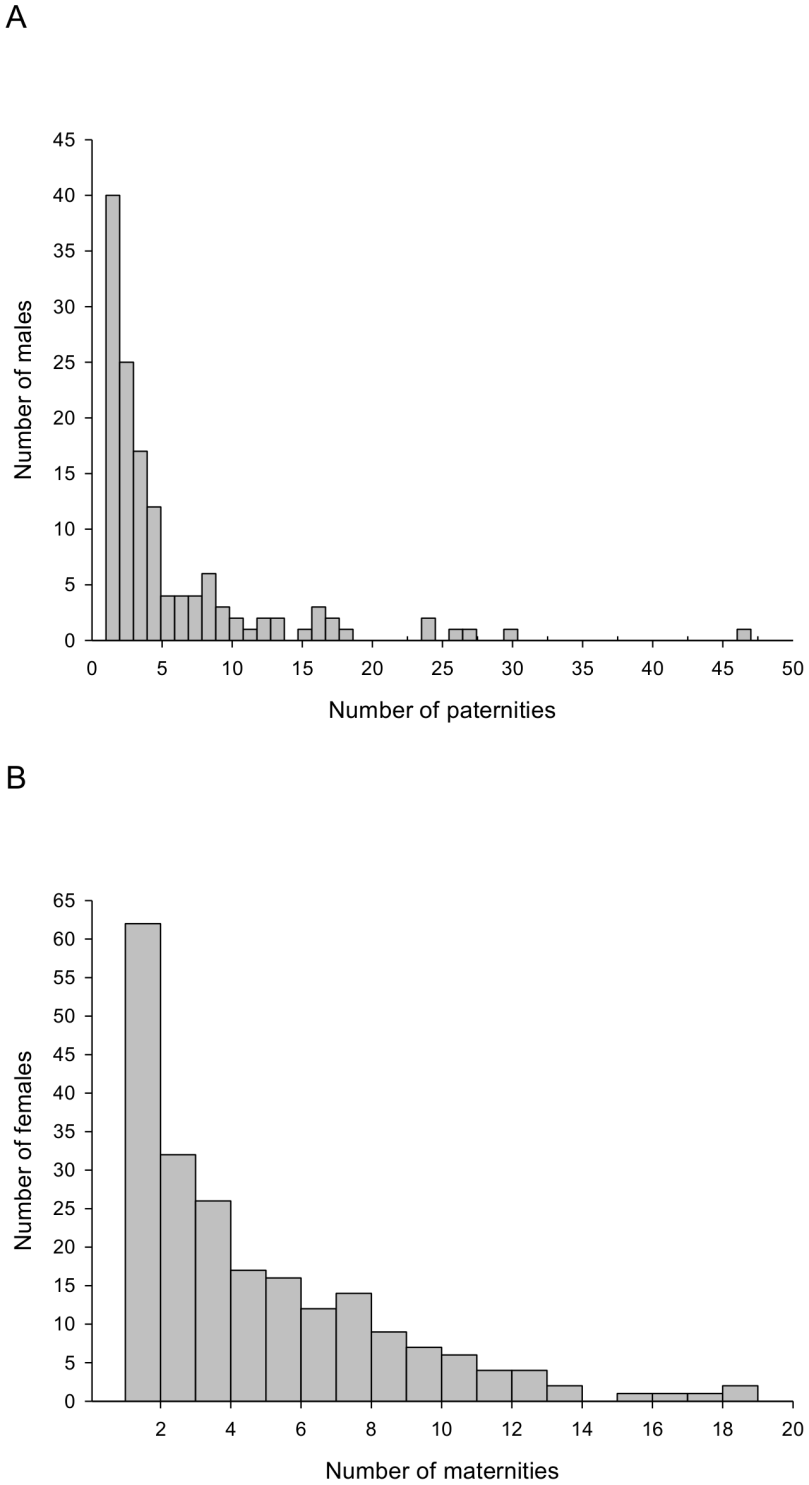
The distribution of reproductive success among greater horseshoe bats. The distribution of paternities and maternities awarded at >80% confidence to A) 135 breeding males, and B) 216 breeding females respectively, in the Woodchester Mansion greater horseshoe bat population over a period of 19 years (1993–2011).

The 710 paternities assigned during the period between 1993 and 2011 were shared among 135 individual males. Annual reproductive success ranged from one to seven pups, with one individual male fathering 47 pups during the 19 years and another five individual males fathering over 20 pups each during the same period. Together these six males sired 178 pups, representing almost one fifth of all offspring born at the Woodchester maternity roost between 1993 and 2011, and 28% of all pups assigned fathers during this period (Figure 1A). Annual reproductive skew among breeders (*B* index) ranged from −0.0388 to 0.017 (Table 1), although *B* index values were not significant in any single year. Likewise, we did not find significant skew between breeding males of the same age. When all 19 cohorts were pooled, however, we detected significant skew among breeders (*B* index = 0.0047, P<0.001, *n* = 135), due to the repeated success of some individuals.

**Figure 2:**
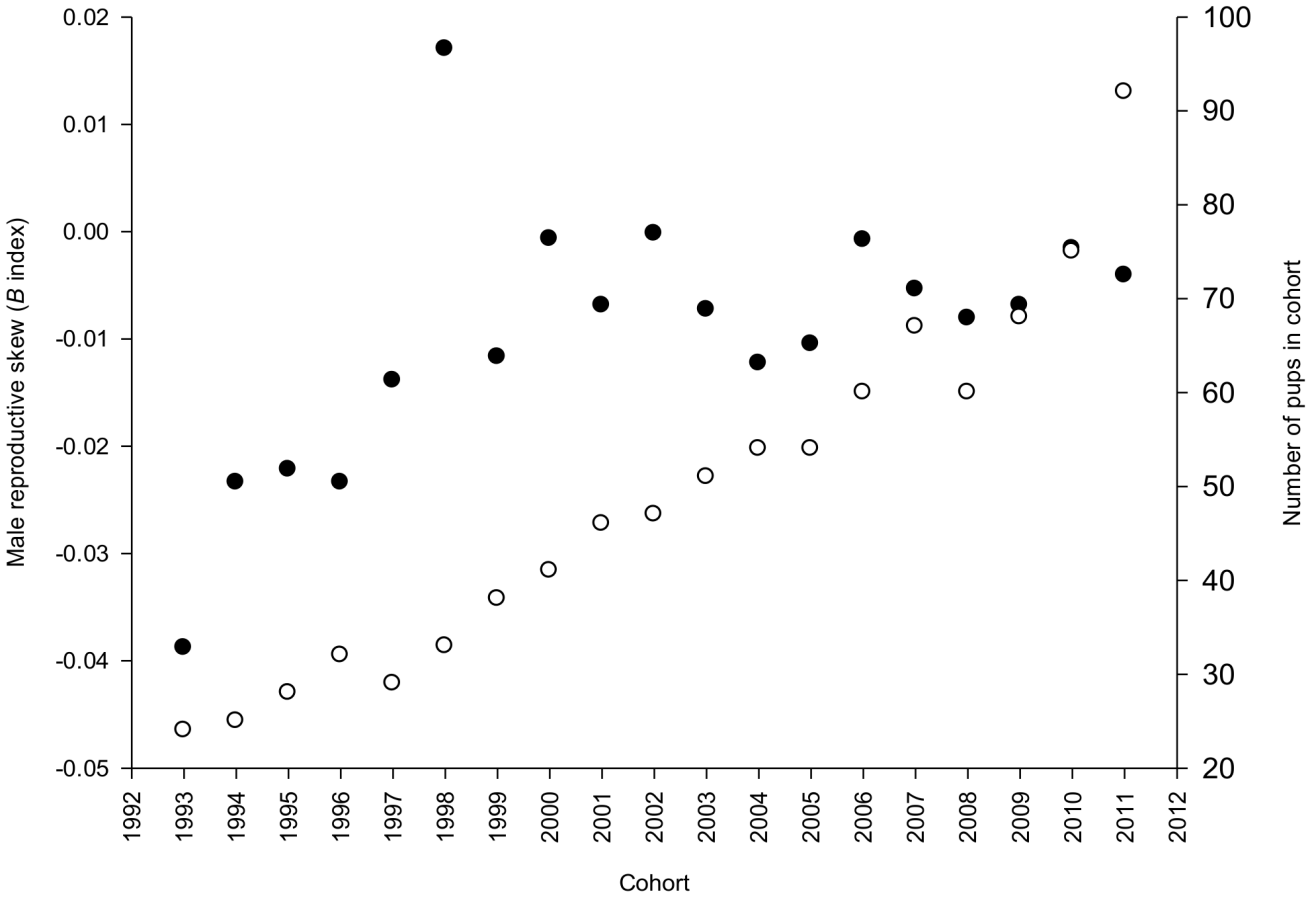
Male reproductive skew and number of pups born between 1993 and 2011. Changes in male reproductive skew (filled circles) and cohort size (open circles) through time in the Woodchester Mansion greater horseshoe bat population. Because adult females maximally produce one offspring per year, the number of pups born is a proxy for the colony size, which in turn reflects the population size.

Although skew was not significant within years, it increased in magnitude over time (t = 2.594, P = 0.0190, *n* = 19), alongside an increase in overall population size (t = 16.535, P<0.0001, *n* = 19). A plot of cohort size against skew revealed a positive trend, illustrating that male reproductive skew among breeding males has tended to increase overall within the population between 1993 to 2011 (Figure 2). However, while the population has continued to grow throughout this period, skew has stabilised since 2000 (Figure 2).

**Figure 3:**
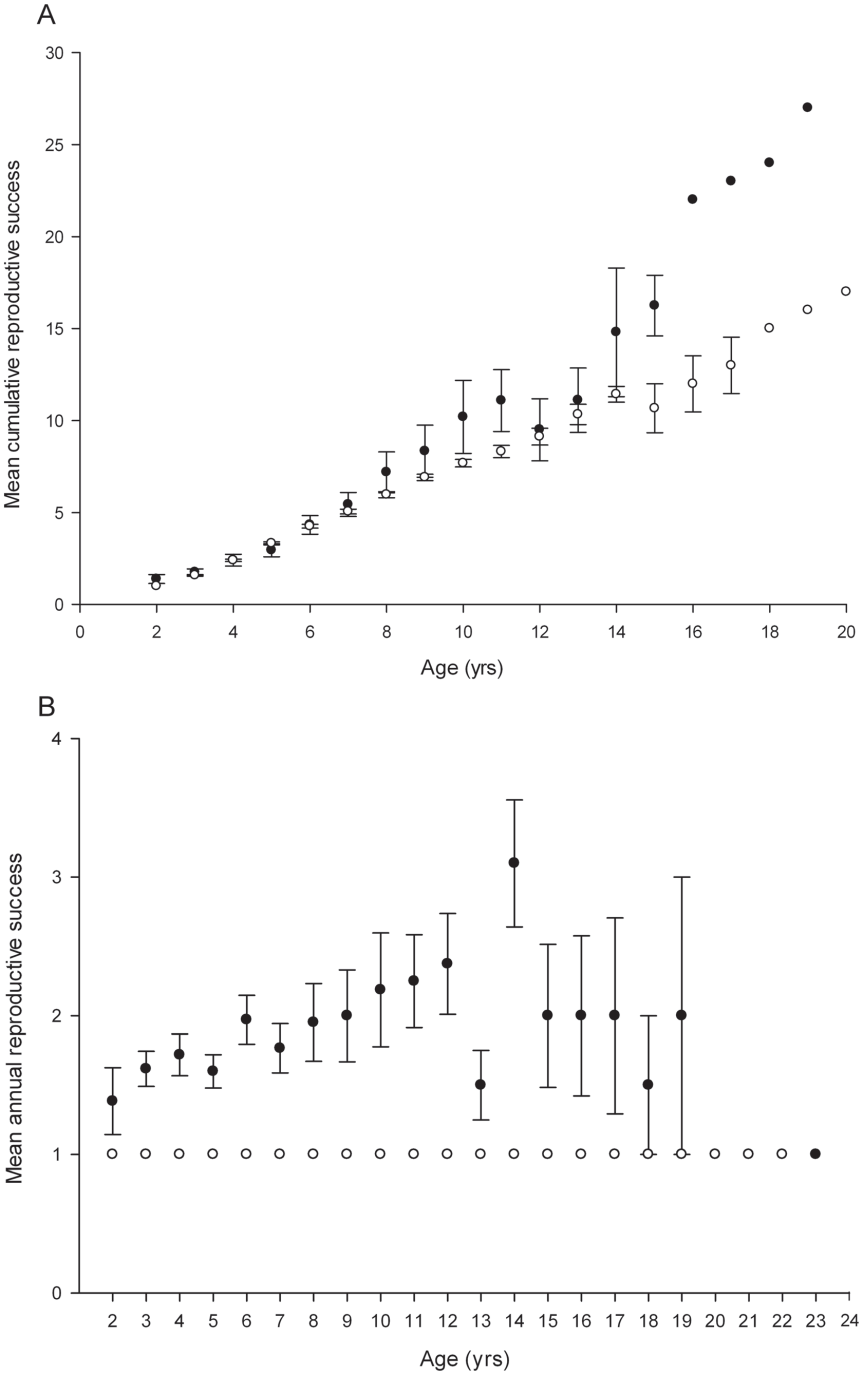
Age and reproductive success in breeding greater horseshoe bats. A) The mean cumulative reproductive success of breeding males (filled circles) and females (open circles), with standard errors, at a given age. This figure only includes data from bats born in or after 1991; we did not include bats born before 1991 because they might have had additional pups prior to 1993. B) The mean annual reproductive success of breeding male (filled circles) and female (open circles) bats in a single year, with standard errors, at a given age. Mean annual female reproductive success at any given age is always 1 as females can only have one pup each year.

While cumulative male paternity success increased consistently with age (filled circles in Figure 3A), a plot of annual paternity success of breeding males against age revealed a quadratic trend, with individual reproductive output increasing steadily from the age of two years to 12 years, but declining after 14 years (filled circles in Figure 3B). To test the significance of this trend, we developed a GLMM in which age was fitted first as a simple term and then also as a quadratic term, with individual identity fitted as a random effect. Age had a significant effect on annual paternity success (*n* = 629, Z = 8.63, P<0.0001) after variance among individuals and years was taken into account (Table 2). Model fit improved significantly when we added age as a quadratic term (log-likelihood = −710.38 versus log-likelihood = −744.65 respectively, χ^2^ test = 68.54, d.f. = 1, P<0.0001) (Table 2).

**Table 2:** Determinants of male and female reproductive success.

		*n*	Age (yrs)		Age^2^		Forearm (mm)		Heterozygosity		Log-likelihood
			Z value	P value	Z value	P value	Z value	P value	Z value	P value	
**Males**	M1	629	8.63	<2e-16***							−744.65
	M2	629	9.54	<2e-16***	−7.59	3.2e-14***					−710.38
	**M3**	**629**	**9.52**	**<2e-16** ***	**−7.53**	**4.9e-14** ***	**2.56**	**0.0105** *			**−707.188**
	M4	629	9.52	<2e-16***	−7.54	4.8e-14***	2.56	0.0105*	−0.99	0.320	−706.69
**Females**	M1	766	4.42	1e-05***							−368.20
	M2	766	6.34	2.26e-10***	−5.39	7.23e-08***					−355.50
	M3	766	6.28	3.43e-10***	−5.42	6.04e-08***	1.02	0.309			−357.40
	**M4**	**766**	**6.22**	**4.94e-10** ***	**−5.20**	**2.07e-07** ***			**2.07**	**0.038** *	**−353.50**

Effect size and significance of each fixed variable added to a general linear mixed model built to describe male and female reproductive success respectively in the Woodchester Mansion greater horseshoe population. MX denotes model number, *n* denotes sample size, na ‘not applicable’,

*** denotes an effect significant at the 0.001 level and

* an effect significant at the 0.05 level. The best model for each sex, which contained only explanatory variables significant at the 0.05 level, was selected using the log-likelihood values of respective models and is highlighted in bold.

**Figure 4:**
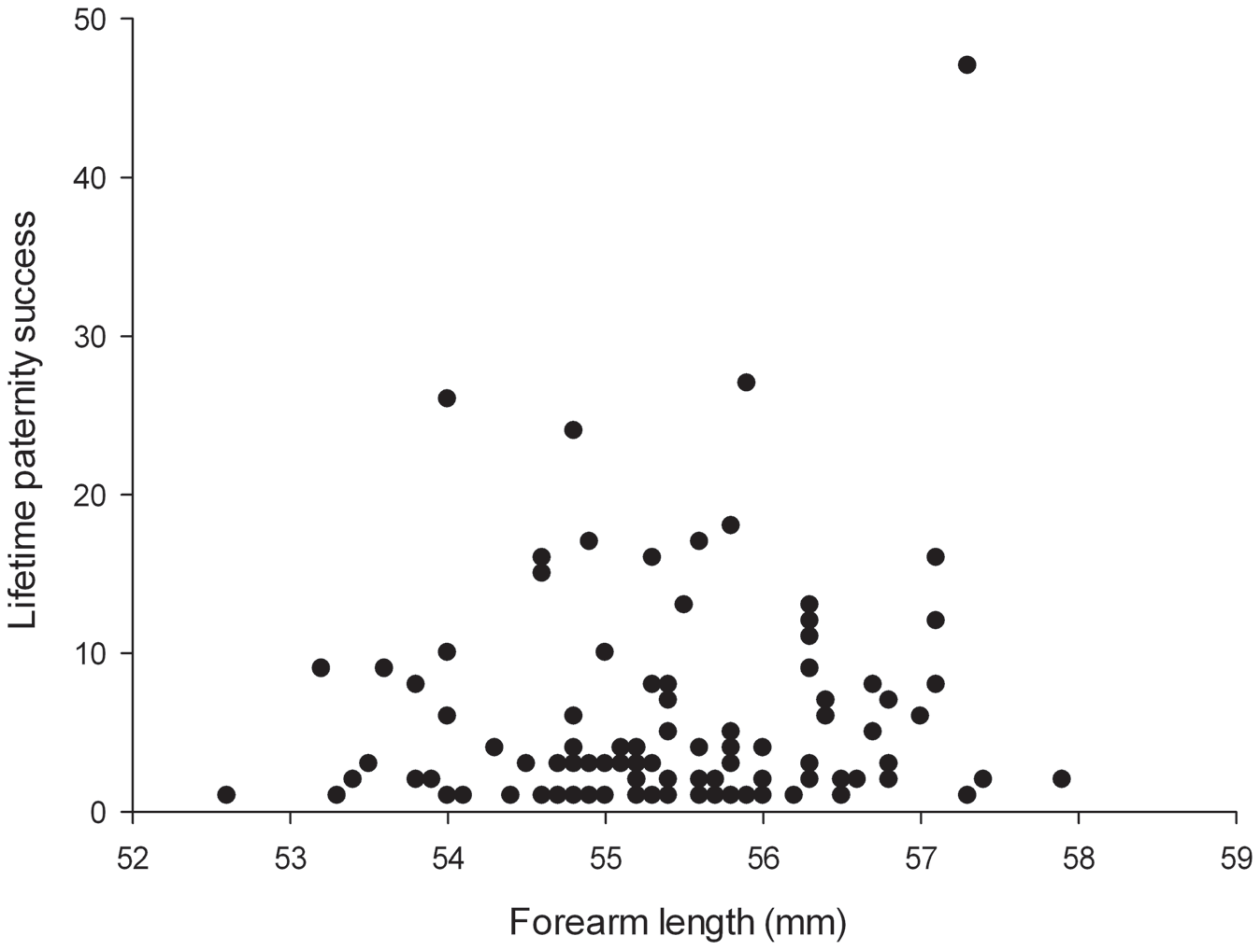
Male reproductive success and forearm length. The relationship between forearm length and paternity success among breeding males between 1993 and 2011; larger males had greater annual reproductive success. In addition, male forearm length significantly and positively influenced total male reproductive success over the 19 year period between 1993 and 2011.

Large standard errors around the mean paternity success of males at a given age indicate that not all variation in annual success was due to age-effects. Adding individual forearm length to the model improved model fit further (log-likelihood = −707.19 versus log-likelihood = −710.38, χ^2^ test = 6.38, d.f. = 1, P = 0.012) (Table 2), implying that male size has a significant effect on paternity success and larger males have more offspring (*n* = 629, Z = 2.56, P = 0.011) (Table 2, for data see Figure 4). This relationship remained significant following the removal of the apparent outlier (see Figure 4) from the data set (*n* = 612, Z = 2.32, P = 0.020). In addition, male forearm length significantly and positively influenced total male reproductive success over the 19 year period between 1993 and 2011 (*n* = 189, Z = 4.165, P<0.0001) (Table 3). We did not, however, find a significant difference in the forearm lengths of male breeders and male non-breeders (*n* = 189, Z = 1.059, P = 0.290) (Table 4).

**Table 3:** The effect of forearm length and genetic heterozygosity on male and female reproductive success.

	*n*	Breeding period (yrs)		Forearm (mm)		Heterozygosity	
		Z value	P value	Z value	P value	Z value	P value
**Males**	189	26.135	<2.00e-16***	4.165	3.12e-05***	0.302	0.763
**Females**	222	24.067	<2.00e-16***	1.415	0.157	2.127	0.033*

The effect of forearm length and genetic heterozygosity on male and female reproductive success respectively, at Woodchester Mansion, over the period 1993–2011. *n* denotes sample size,

*** denotes an effect significant at the 0.001 level,

** an effect significant at the 0.01 level and

* an effect significant at the 0.05 level.

**Table 4:** Comparing the mean forearm length and heterozygosity of breeding and non-breeding male and female bats.

	Forearm length (mm)								Heterozygosity							
	Breeding bats			Non-breeding bats					Breeding bats			Non-breeding bats				
	*n*	Mean	S.D.	n	Mean	S.D.	Z value	P value	*n*	Mean	S.D.	*n*	Mean	S.D.	Z value	P value
**Males**	82	55.52	0.950	107	55.37	0.964	1.059	0.290	82	1.003	0.150	107	1.019	0.178	−0.625	0.532
**Females**	166	56.40	1.015	56	56.11	1.160	1.771	0.077	166	1.005	0.152	56	1.005	0.165	0.030	0.976

Comparing the mean forearm length (mm) and heterozygosity of breeding and non-breeding male and female bats respectively. *n* denotes sample size.

Finally, individual heterozygosity did not significantly influence the division of male reproductive success among males (Table 2, Table 3). Nor was there a significant difference between the heterozygosity scores of breeding and non-breeding males (Table 4).

### Female reproductive success

The 919 maternities assigned during the period between 1993 and 2011 were shared among 216 individual females. Female greater horseshoe bats showed considerable variation in total reproductive success among breeding females across the 19 year period, which ranged from one to eighteen pups (Figure 1B). This apparent skew was not significant when maternity data for all 19 cohorts were pooled and reproductive success was corrected for the number of years each female was breeding during the 19 year period (*B* index = −0.0009, P = 1, *n* = 216). Because females can only produce a maximum of one pup each year so there was no variance in reproductive success (and therefore no reproductive skew) among breeding individuals within years or among breeders of the same age (Figure 3B).

The plot of the maternity success of breeding females against age revealed that, like males, cumulative individual reproductive output increased from the age of two to 20 years (Figure 3A) albeit at a steadier rate than male reproductive success. A GLMM in which age was fitted first as a simple term and then also as a quadratic term, with individual identity and year of birth fitted as random effects, confirmed that age had a significant effect on maternity success (*n* = 766, Z = 4.42, P<0.0001) after variance among individuals and years was taken into account (Table 2). Model fit improved significantly when we added age as a quadratic term as well as a simple term (log-likelihood = −355.50 versus log-likelihood = −368.20 respectively, χ^2^ test = 25.40, d.f. = 1, P<0.0001) (Table 2).

**Figure 5:**
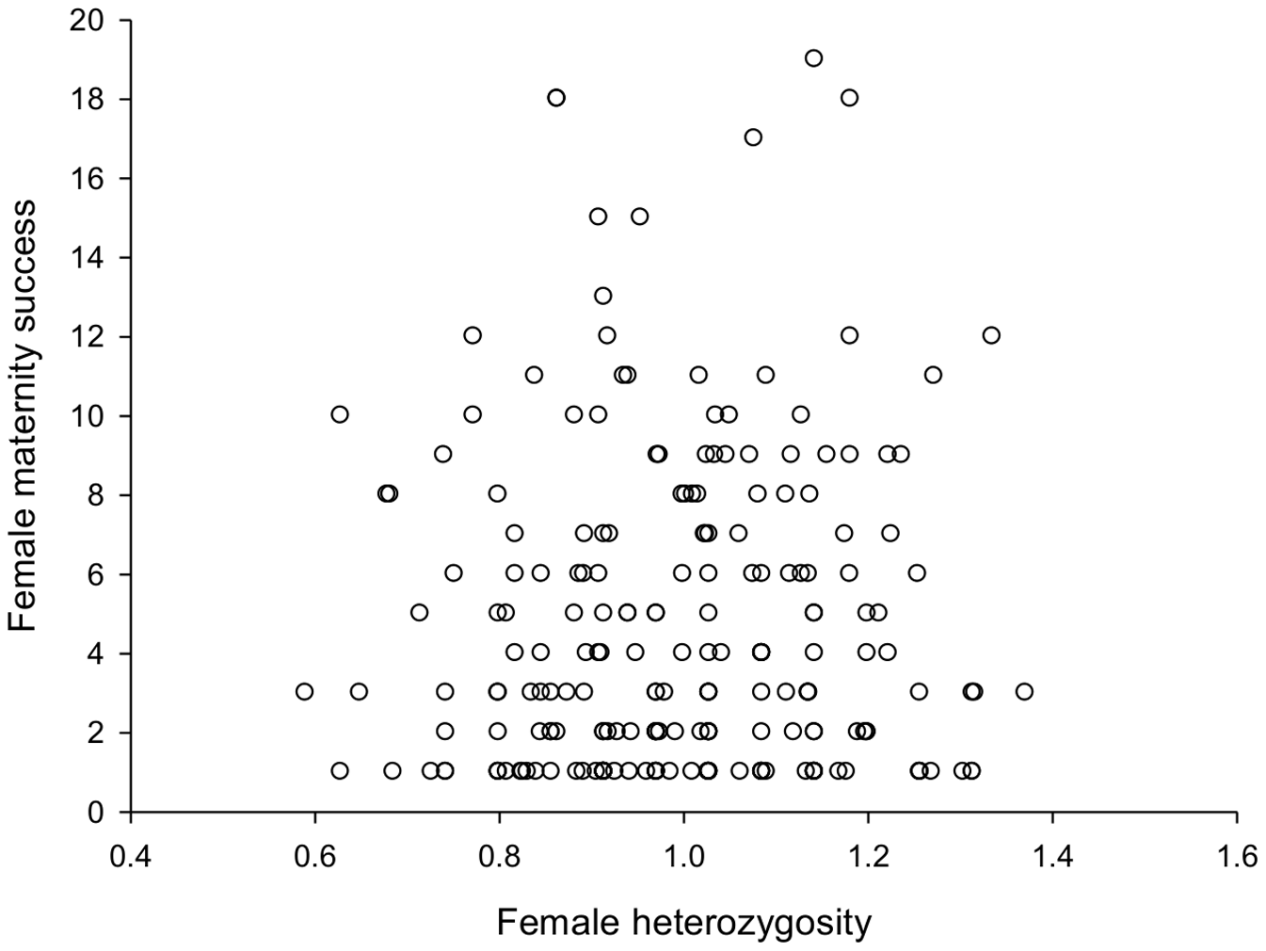
Female reproductive success and heterozygosity. The relationship between heterozygosity and maternity success among breeding females between 1993 and 2011; more heterozygous females had greater annual reproductive success. Heterozygosity was also a significant predictor of, and positively correlated with, the total number of pups a breeding female gave birth to between 1993 and 2011.

As for males, large standard errors around the maternity success of females at a given age indicated that not all variation in annual success was due to age-effects, however, adding individual forearm length to the model did not improve model fit, implying that female size did not have a significant influence on maternity success (Table 2). In addition, breeding females did not have significantly larger forearms than non-breeding females (Table 4). In contrast, heterozygosity influenced the division of reproductive success among breeding females. Adding individual heterozygosity to the mixed model significantly improved model fit (log-likelihood = −353.50 versus log-likelihood = −355.50, χ^2^ test = 4.00, d.f. = 1, P = 0.046), and implied that more heterozygous females produced more offspring (*n* = 766, Z = 2.07, P = 0.038) (Table 2, Figure 5). Consistent with this result we also found heterozygosity to be a significant predictor of, and positively correlated with, the total number of pups a breeding female gave birth to between 1993 and 2011 (*n* = 222, Z = 2.127, P = 0.0334) (Table 3). We did not find a significant difference in heterozygosity between breeding and non-breeding females, however (*n* = 222, Z = 0.030, P = 0.976) (Table 4).

## Discussion

We studied the determinants of male and female reproductive success in wild greater horseshoe bats (*Rhinolophus ferrumequinum*) by examining patterns of parentage over a 19 year period between 1993 and 2011. Based on a likelihood method, mothers were assigned to 99.5% and fathers to 76.8% of 924 pups born into the Woodchester population.

### Determinants of reproductive success in males and females

Male reproductive success was influenced by age, showing a similar trend to those reported for other long-lived mammals [58], [59] and supporting earlier findings from the same population based on a smaller sample size [38]. In addition, here we show for the first time that male success is also positively related to body size, with larger males siring more pups, a trend more commonly seen in taxa with male-biased size dimorphism. This observed effect of size, which was unrelated to age, contrasts with another polygynous bat species, *Saccopteryx bilineata*, in which small males gain more paternities, probably because of their greater speed and agility in aerial courtships and defence manoeuvres for protecting female harems [60]. Similarly, in some raptors and owls that show reversed sexual size dimorphism (RSD) small males gain higher reproductive success because they are able to expend less energy whilst foraging [61].

The advantage of large body size in greater horseshoe bats, which are not known to court aerially, suggests that males compete for access to females, and that larger males win these contests more often. However, intra-sexual contests between male greater horseshoe bats over females have not been observed; instead it is thought females actively seek out and choose males in their underground mating territories. Consequently females may choose mates either on the basis of their individual traits or on the basis of the quality of their territories, which might differ in their proximity to the maternity roost, suitability as a hibernaculum or the quality of the surrounding habitat for foraging [40]. Taking these observations into account, the mating system of this species probably contains elements of both female choice and male-male competition, whereby any advantage of larger size to males comes from indirect competition for females via direct competition for mating sites. Certainly individual sites are normally only occupied by a single male, who is replaced quickly on disappearance, implying strong competition for sites, and we have identified several important sites where the resident male has consistently high paternity success [38].

Breeding success among females was also found to be related to age. Specifically, most females produced one pup per year from their age of first breeding (normally 2 or 3 years) and continued until around 12 years old, after which breeding frequency was less consistent with occasional non-breeding years [41]. A decrease in reproductive output with age in animals is commonly attributed to senescence associated with old age per se, and/or a decline in fertility or competitive ability [8], [62]. However, as with paternity, not all variation in annual maternity success was explained by age-effects; we also found a positive relationship with individual heterozygosity. Heterozygosity-fitness correlations have been well studied in wild populations and small but significant positive effects for life-history, morphological, and physiological traits have been consistently shown [63]. Two alternative hypotheses exist to account for associations between neutral markers and fitness traits; the ‘general effect’ hypothesis, which theorises that heterozygosity measured at a suite of loci reflects genome-wide diversity, and the ‘local effect’ hypothesis, which suggests apparent fitness can increase with increasing heterozygosity at marker loci because they are in linkage disequilibrium with loci affecting fitness [63]. The Woodchester population of greater horseshoe bats suffered a population bottleneck due to poor weather in 1986–1987 and since that time the population has been increasing from a small initial size. Certainly over the beginning of this period, which predated sampling, it is plausible that genetic diversity was reduced and the potential for consanguineous matings was high. Therefore, females with higher heterozygosity would have likely been less inbred. Inbreeding reduces individual multilocus heterozygosity, increasing the risk of expressing recessive deleterious alleles and decreasing the occurrence of beneficial over-dominant effects (inbreeding depression) [64]. In this situation a positive relationship between multilocus heterozygosity and fitness is expected [9]. Offering further support for this argument, more outbred females in the Woodchester population have already been shown to have better survival [42], which would in turn increase their chance of having greater reproductive success.

Female reproductive success was surprisingly not found to be influenced by size based on the data analysed, in spite of the fact that females are the larger sex and that males show size-related paternity and greater variance in reproductive success. One possible explanation is that females have to be above a threshold size to breed, partly because they have to have a wing area large enough to be able to fly while carrying heavy loads during pregnancy [24], [65]. This larger size may be suboptimal for foraging, so the selective pressure for males to be larger in order to gain greater paternity success is counterbalanced by a selective pressure against growing too large; hence males tend to be smaller. There is some evidence that this may be the case; in poor weather years, when there are fewer insects flying so less food available, pup sex ratios tend to be biased towards males [66], which implies that mothers may be unable to support larger, more costly female offspring to term.

### Breeding skew

Parentage analyses revealed a polygynous breeding system with significant long-term reproductive skew among breeding males, but not among breeding females. Among individual males, a steady increase in mean cumulative reproductive success was detected over the study period, which was also corroborated by the highly significant paternity skew estimated from the pooled paternity assignments based on 19 cohorts. Together these data provide good evidence that while paternity skew within any one year was not sufficiently strong to be deemed significant, the subtle differences observed among males arose from intrinsic differences among individuals rather than by stochastic effects [38]. Indeed a plot of annual skew across the study duration showed an increase from 1993 to around 2000, coinciding with the overall upward trend in the numbers of the study population. During this period, therefore, a small minority of males in this population appear to have increased their share of mates (and thus paternities) despite the presence of ever growing numbers of other males that could potentially breed. Our findings from this period thus add some weight to predictions from simulations that increasing population density, and thus the intensity of sexual selection, will favour high quality males [25]. On the other hand, we also noted that annual skew has appeared to trail off over the past nine years, in spite of the continued demographic growth. This is intriguing because it would appear to suggest that there is a natural limit to the number of females that a male can easily breed with in any one year, in this case around four to six individuals, perhaps because of his need to defend his territory from other males. As such, our data also support observations that breeding skew does not always rise with available partners due to the problems of guarding resources [25], [26].

To date little or no male reproductive skew has been reported in other temperate bat species. Although this has been attributed to aspects of mating behaviour that prevent mate guarding [67], our data also highlight a need for long-term data in order to uncover patterns of variance in paternity success. Evidence of polygyny in bats is arguably more easily detected in tropical non-hibernating species, where males defend small groups of females for longer time-periods [68]. Our values of paternity skew are similar to those obtained for rhesus macaques (*Macaca mulatta*), a species with male-biased size dimorphism in which reproductive success correlates in part with dominance rank [69], but lower than those obtained for white-faced capuchins (*Cebus capucinus*), another species with male-biased size dimorphism but one that lives in groups with a clear alpha male who can retain tenure and monopolise breeding for many successive years [70]. These comparisons imply that there could be a weak hierarchy among Woodchester males, with males at the top of the hierarchy procuring more matings. A hierarchy does not have to imply direct competition between males, however; male position could be determined by female mating preferences for males with certain characteristics or mating territories.

In contrast to males, analyses of a long-term dataset provided no evidence that maternity success was significantly skewed among breeding females. Female reproductive skew is most commonly reported within cooperatively breeding species [21], but has also been documented in populations of mammal species that, like the greater horseshoe bat, exhibit reversed sexual size dimorphism, such as the spotted hyena in which there is aggressive competition for males and female reproductive success is strongly correlated with social rank [15]. However, a lack of evidence of social hierarchy among female greater horseshoe bats, as well as the lack of skew documented among breeders and the observation that female reproductive success does not relate to body size. all suggest that there is little or no intra-sexual competition among females for males in this population. Indeed, almost all females that reach breeding age (≥2 years) do successfully breed. Our results are more consistent with male-dominated polygynous breeding systems, where female success is less dependent on body size [71]. The fact that almost all females, but only a third of males, breed within the Woodchester population, despite a relatively even sex ratio, supports the commonly held theory that females are more selective when it comes to choosing mates than males [64].

## Conclusions

The remarkable male reproductive skew demonstrated within this population clearly demonstrates that a lack of male-biased size dimorphism should not be considered evidence for reduced sexual competition. Indeed, the paternity success of males in this population was significantly influenced by body size, as is almost always found in species with polygynous breeding systems that exhibit male-biased sexual size dimorphism and are characterised by strong intra-sexual competition for mates. The reproductive success of male and female bats within the population was not dependent upon the same traits, however, implying that the two sexes are subject to different selective pressures. More studies from a range of taxa are needed to identify the traits that enhance fecundity in species without male-biased size dimorphism. Such data would contribute greatly to our understanding of sexual selection and the evolution of polygynous breeding systems.

## Supporting Information

Table S1 Characteristics of 38 microsatellite loci, used in parentage analysis, when amplified in greater horseshoe bats.
(DOCX)
