# Supplementary material for: Determinants and Patterns of Reproductive Success in the Greater Horseshoe Bat during a Population Recovery
Source: PLoS One. 2014 Feb 13;9(2):e87199. doi: 10.1371/journal.pone.0087199 (PMC3923748; doi:10.1371/journal.pone.0087199)
Supplement: Table S1 — Characteristics of 38 microsatellite loci, used in parentage analysis, when amplified in greater horseshoe bats. (DOCX) [file pone.0087199.s001.docx]

Table S1 Characteristics of 38 microsatellite loci, used in parentage analysis, when amplified in greater horseshoe bats ‡.

| **Species** | **Microsatellite** | **Accession No.** | **No. of alleles** | **Product length (bp)** | **Reference** |  |
| --- | --- | --- | --- | --- | --- | --- |
| *R. affinis* | A26 | EU737082 | 6 | 187-212 | Mao *et al.* (2009) [[1](#_ENREF_1)] |  |
| *R. affinis* | B63 | EU737086 | 6 | 191-203 | Mao *et al.* (2009) [[1](#_ENREF_1)] |  |
| *R. affinis* | D6 | EU737088 | 5 | 207-217 | Mao *et al.* (2009) [[1](#_ENREF_1)] |  |
| *R. affinis* | E7 | EU737089 | 9 | 274-295 | Mao *et al.* (2009) [[1](#_ENREF_1)] |  |
| *R. affinis* | E95 | EU737094 | 5 | 111-129 | Mao *et al.* (2009) [[1](#_ENREF_1)] |  |
| *R. ferrumequinum* | Rferr01 | AF160200 | 4 | 122-130 | Rossiter *et al.* (1999) [[2](#_ENREF_2)] |  |
| *R. ferrumequinum* | Rferr03 | AF160202 | 10 | 213-240 | Rossiter *et al.* (1999) [[2](#_ENREF_2)] |  |
| *R. ferrumequinum* | Rferr06 | AF160205 | 18 | 198-226 | Rossiter *et al.* (1999) [[2](#_ENREF_2)] |  |
| *R. ferrumequinum* | Rferr08 | AF160207 | 6 | 140-150 | Rossiter *et al.* (1999) [[2](#_ENREF_2)] |  |
| *R. ferrumequinum* | Rferr09 | AF160208 | 9 | 122-140 | Rossiter *et al.* (1999) [[2](#_ENREF_2)] |  |
| *R. ferrumequinum* | Rferr11 | AF160210 | 10 | 182-204 | Rossiter *et al.* (1999) [[2](#_ENREF_2)] |  |
| *R. ferrumequinum* | Rferr12 | AF160211 | 5 | 215-229 | Rossiter *et al.* (1999) [[2](#_ENREF_2)] |  |
| *R. ferrumequinum* | Rferr13 | AJ560694 | 7 | 89-104 | Dawson *et al.* (2004) [[3](#_ENREF_3)] |  |
| *R. ferrumequinum* | Rferr15 | AJ560695 | 3 | 234-238 | Dawson *et al.* (2004) [[3](#_ENREF_3)] |  |
| *R. ferrumequinum* | Rferr16 | AJ560696 | 7 | 192-205 | Dawson *et al.* (2004) [[3](#_ENREF_3)] |  |
| *R. ferrumequinum* | Rferr17 | AJ560697 | 8 | 276-306 | Dawson *et al.* (2004) [[3](#_ENREF_3)] |  |
| *R. ferrumequinum* | Rferr18 | AJ560698 | 2 | 196-202 | Dawson *et al.* (2004) [[3](#_ENREF_3)] |  |
| *R. ferrumequinum* | Rferr19 | AJ560702 | 3 | 199-205 | Dawson *et al.* (2004) [[3](#_ENREF_3)] |  |
| *R. ferrumequinum* | Rferr20 | AJ560703 | 2 | 157-158 | Dawson *et al.* (2004) [[3](#_ENREF_3)] |  |
| *R. ferrumequinum* | Rferr22 | AJ560704 | 7 | 184-197 | Dawson *et al.* (2004) [[3](#_ENREF_3)] |  |
| *R. ferrumequinum* | Rferr24 | AJ560706 | 3 | 376-380 | Dawson *et al.* (2004) [[3](#_ENREF_3)] |  |
| *R. ferrumequinum* | Rferr25 | AJ560708 | 12 | 296-388 | Dawson *et al.* (2004) [[3](#_ENREF_3)] |  |
| *R. ferrumequinum* | Rferr27 | AJ560710 | 6 | 161-183 | Dawson *et al.* (2004) [[3](#_ENREF_3)] |  |
| *R. ferrumequinum* | Rferr28 | AJ560711 | 3 | 146-155 | Dawson *et al.* (2004) [[3](#_ENREF_3)] |  |
| *R. ferrumequinum* | Rferr29 | AJ560712 | 19 | 276-354 | Dawson *et al.* (2004) [[3](#_ENREF_3)] |  |
| *R. ferrumequinum* | Rferr30 | AJ560713 | 12 | 243-290 | Dawson *et al.* (2004) [[3](#_ENREF_3)] |  |
| *R. hipposideros* | RHA4 | NA | 5 | 270-280 | NA |  |
| *R. hipposideros* | RHA8 | JF750631 | 9 | 146-177 | NA |  |
|  |  |  |  |  |  |  |
| *R. hipposideros* | RHA102 | NA | 5 | 286-299 | NA |  |
| *R. hipposideros* | RHA104 | JF750633 | 6 | 272-299 | NA |  |
| *R. hipposideros* | RHA101 | JF750632 | 5 | 143-151 | NA |  |
| *R. hipposideros* | RHA118 | JF750636 | 3 | 217-231 | NA |  |
| *R. pusillus* | Rhpu-A4 | EF423560 | 4 | 190-202 | Hua *et al.* (2009) [[4](#_ENREF_4)] |  |
| *R. pusillus* | Rhpu-E11A | EU559248 | 8 | 99-141 | Hua *et al.* (2009) [[4](#_ENREF_4)] |  |
| *R. pusillus* | Rhpu-H3 | EF423570 | 12 | 280-320 | Hua *et al.* (2009) [[4](#_ENREF_4)] |  |
| *R. pusillus* | Rhpu-PD3 | EF423565 | 5 | 212-221 | Hua *et al.* (2009) [[4](#_ENREF_4)] |  |
| *R. pusillus* | Rhpu-PH30 | EF423561 | 4 | 175-186 | Hua *et al.* (2009) [[4](#_ENREF_4)] |  |
| *R. pusillus* | Rhpu-PH69A | EU559249 | 5 | 106-119 | Hua *et al.* (2009) [[4](#_ENREF_4)] |  |
| *R. sinicus* | Rhsi-SN80 | EU780430 | 9 | 156-184 | Liu *et al.* (2009) [[5](#_ENREF_5)] |  |
| *R. sinicus* | Rhsi-SN91 | EU780431 | 4 | 222-229 | Liu *et al.* (2009) [[5](#_ENREF_5)] |  |

‡ Upon completion of genotyping, 102 individuals were genotyped at up to 21 loci of the 40 loci listed above (individuals that were sampled prior to 2003, for which we no longer have viable DNA samples), 352 individuals at up to 40 loci (individuals sampled prior to 2003 for which we still have viable DNA samples), and 741 individuals at up to 33 loci (individuals sampled between 2003 and 2011). In reality, many of the pre-2003 individuals are genotyped at more loci than stated above because they were resampled post-2003 and the second sample was genotyped for newly developed loci.

1. Mao XG, Liu Y, Zhou YY, He BB, Zhang SY (2009) Development of 19 polymorphic microsatellite loci for the intermediate horseshoe bat, *Rhinolophus affinis* (Rhinolophidae, Chiroptera). Conservation Genetics 10: 709-711.

2. Rossiter SJ, Burland TM, Jones G, Barratt EM (1999) Characterization of microsatellite loci in the greater horseshoe bat *Rhinolophus ferrumequinum*. Molecular Ecology 8: 1959-1960.

3. Dawson DA, Rossiter SJ, Jones G, Faulkes CG (2004) Microsatellite loci for the greater horseshoe bat, *Rhinolophus ferrumequinum* (Rhinolophidae, Chiroptera) and their cross-utility in 17 other bat species. Molecular Ecology Notes 4: 96-100.

4. Hua PY, Guo TT, Liu WC, Zhang SY, Rossiter S (2009) Isolation and characterization of 13 microsatellite loci in *Rhinolophus pusillus* (least horseshoe bat) with cross-amplification in five related species. Conservation Genetics 10: 597-600.

5. Liu WC, Zhang JS, Hua PY, Zhang SY, Rossiter SJ (2009) Development and characterization of novel microsatellite markers from the Chinese rufous horseshoe bat (*Rhinolophus sinicus*) with cross-species amplification in closely related taxa. Molecular Ecology Resources 9: 183-185.
